# Editorial: Impact of anthropogenic stressors on marine sponge holobiomes

**DOI:** 10.3389/fmicb.2024.1533416

**Published:** 2024-12-13

**Authors:** Nicole Trefault

**Affiliations:** ^1^GEMA Center for Genomics, Ecology and Environment, Universidad Mayor, Santiago, Chile; ^2^Millenium Nucleus in Marine Agronomy of Seaweed Holobionts (MASH), Puerto Montt, Chile; ^3^FONDAP Center IDEAL- Dynamics of High Latitude Marine Ecosystem, Valdivia, Chile

**Keywords:** anthropogenic stressors, sponge holobiome, symbiotic microorganisms, human activities, sponge holobionts

Oceans around the globe are experiencing faster and increasing threats with unprecedented consequences, with human activities being one of the main drivers of ocean deterioration worldwide. Anthropogenic stressors have profoundly impacted marine environments in the last centuries, leading to monumental environmental disasters and increasing consequences of climate change.

Main human-induced changes that adversely affect marine ecosystems include industrial and agricultural runoff pollution, overfishing, habitat destruction from coastal development, seabed mining, and climate change, among others. Runoff pollution leads to chemical contamination and eutrophication, which can deplete oxygen levels and harm marine life. Overfishing disrupts food webs and reduces biodiversity, while habitat destruction from coastal development and seabed mining can lead to the loss of crucial breeding and feeding grounds. Climate change, driven by greenhouse gas emissions, also alters ocean temperatures and acidity, threatening coral reefs and other sensitive marine habitats. Combined, these factors significantly impact the health, productivity, and diversity of marine ecosystems, calling for urgent conservation and management efforts to mitigate their effects.

Symbiotic microorganisms can confer increased resilience to marine holobionts, the ensemble formed by multiple living beings in close relationship with one another and functioning as a unit. This element has not been sufficiently considered as part of the challenges facing marine ecosystems as a result of anthropogenic stressors and climate change.

We already know microbial symbioses have deep implications for the evolution and fitness of all organisms on Earth. However, the impact of stressors of human origin, including pesticides, oils, plastics, antibiotics, trace metals, temperature and pH increases, and so on, has not been adequately addressed in the context of microbial interactions and symbiosis.

Particularly, marine sponges stand out for their incredible filtration capacity and interaction mechanisms with the environment. Due to its early appearance in our evolutionary history, they represent a basal model of animal symbiosis (Pita et al., 2018). Their unique morphology and biological functions provide essential habitats for a myriad of microorganisms, forming complex holobionts that contribute to nutrient cycling, habitat formation, and marine biodiversity. As threats to marine environments increase due to climate change, pollution, and overexploitation, understanding the impact of anthropogenic stressors on the marine sponge holobiome is more critical than ever.

This Research Topic of Frontiers in Microbiology titled “*Impact of anthropogenic stressors on marine sponge holobiome*” aims to illuminate the multifaceted relationships within sponges and their associated microbial communities and how these dynamics shift under human-induced pressures.

In Vad et al. researchers expose *Halichondria panicea* holobionts to oil contaminated seawater, finding an over expression of detoxification and immune response pathways in the host. The sponge holobiont can be exposed to hydrocarbons through the consumption of marine oil snow, but the host and the symbiotic bacteria responses differ. Results indicated that the sponge-associated microbial community is capable of hydrocarbon degradation but without sufficient resilience against oil contaminants, as sponge holobionts exposed to very low concentrations of hydrocarbons died after 7 days of exposure.

De Castro-Fernández et al., studied the effects of elevated seawater temperature on sponge holobionts from several worldwide locations and whether sponges from polar areas are more sensitive to these impacts concerning temperate and tropical species. The authors demonstrated that the core microbiome is maintained in most sponges after heat stress and that sponges from Antarctic waters could be more resilient than tropical and temperate ones. Microbiome community composition and heat stress-induced responses depend on the sponge species, reflecting the host-species specificity.

The group by Carr et al. investigated the incredible potential for degradation of polyester substrates of bacteria isolated from marine sponges. In this study, authors characterize the hydrolysis of PET by a polyesterase previously identified from *Streptomyces* sp. SM14, an isolate of the marine sponge *Haliclona simulans*. The SM14 polyesterase preferred high salt conditions, with significant activity increasing at higher sodium chloride concentrations. This result suggests their potential use in the biological degradation of plastic particles that readily contaminate marine ecosystems and industrial wastewater.

In Dinçtürk et al., the authors explore the mass mortality of wild sponges in the Eastern Mediterranean, finding the impact of three putatively pathogenic *Vibrio* strains, isolated only from tissues of diseased keratose demosponge *Sarcotragus foetidus* sponges. The researchers documented a progressive increase in sea temperature from 1970 to 2021 and a historical maximum of almost 30°C in August 2021, which was coincident with the mass mortality of the keratose sponge, suggesting that elevated sea temperature promoted the proliferation of thermo-dependent pathogenic *Vibrio* species in *S. foetidus*, impacting the course of the sponge disease.

Finally, in the article by Papale et al., the Antarctic demosponge species from the Ross Sea, *Haliclona dancoi* and *Haliclona scotti*, and their bacterial communities were tested for the presence of polychlorobiphenyls, polycyclic aromatic hydrocarbons, and trace metals. Results showed higher amounts of pollutants in the sponge holobionts than in the surrounding sediment and seawater. Interestingly, some bacterial groups were correlated to the occurrence of certain contaminants, suggesting that these bacteria could protect the sponges by transforming pollutants or participating in their excretion and demonstrating the accumulation capability of the sponge holobionts.
